# Supplementary figures and images for: Pathogenic characteristics of an aggregated diarrhea event caused by Plesiomonas shigelloides from stream
Source: PLoS One. 2024 Apr 4;19(4):e0301623. doi: 10.1371/journal.pone.0301623 (PMC10994385; doi:10.1371/journal.pone.0301623)

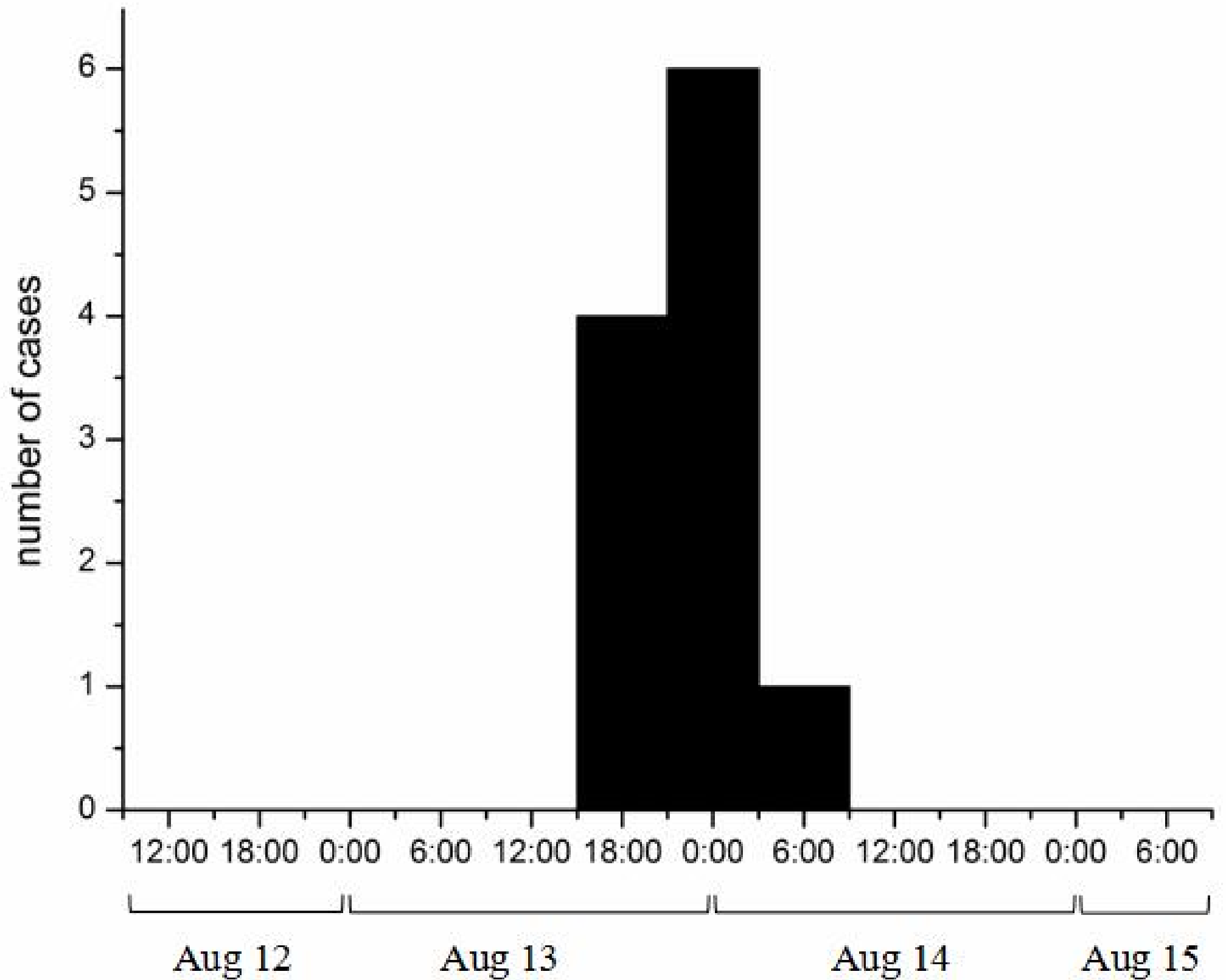

marker

S588

S589

S590

marker

S591

S592

S593

S594

marker

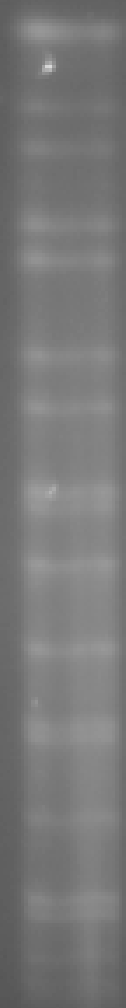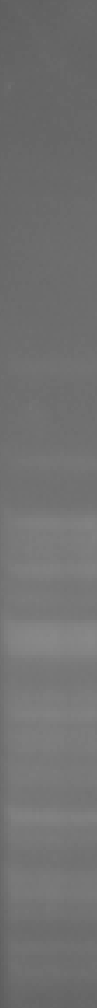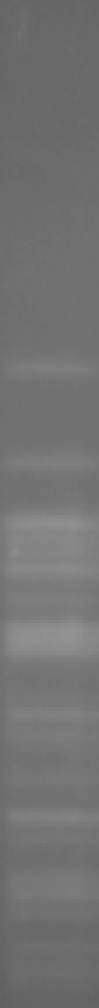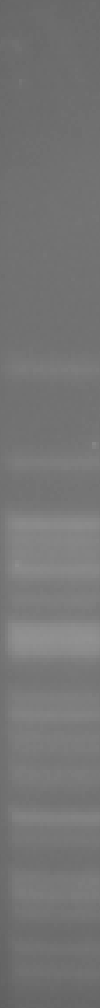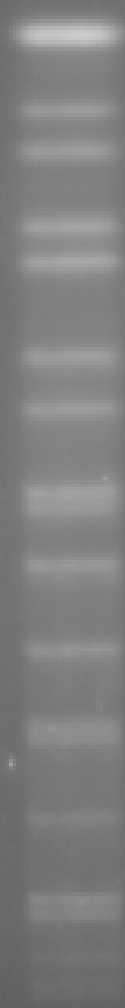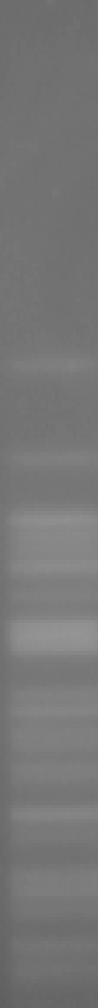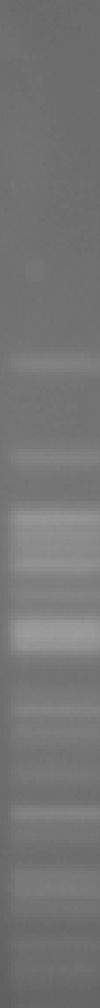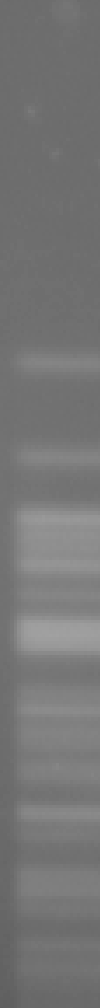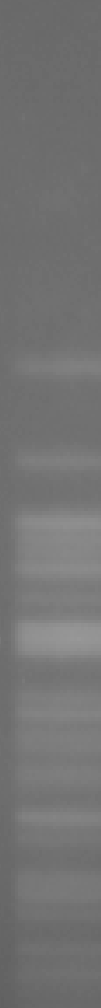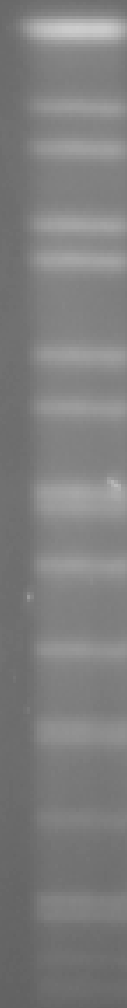

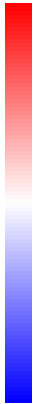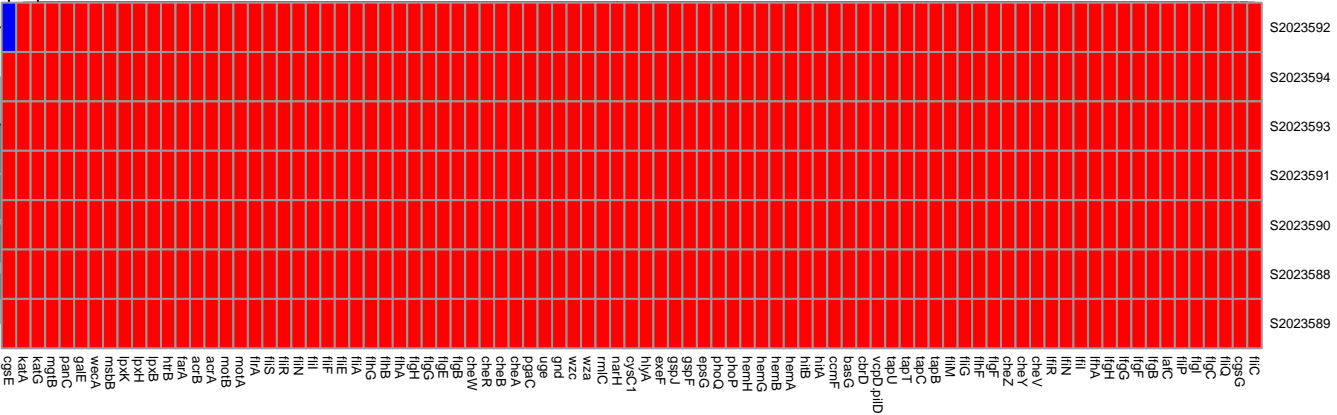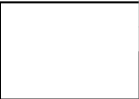

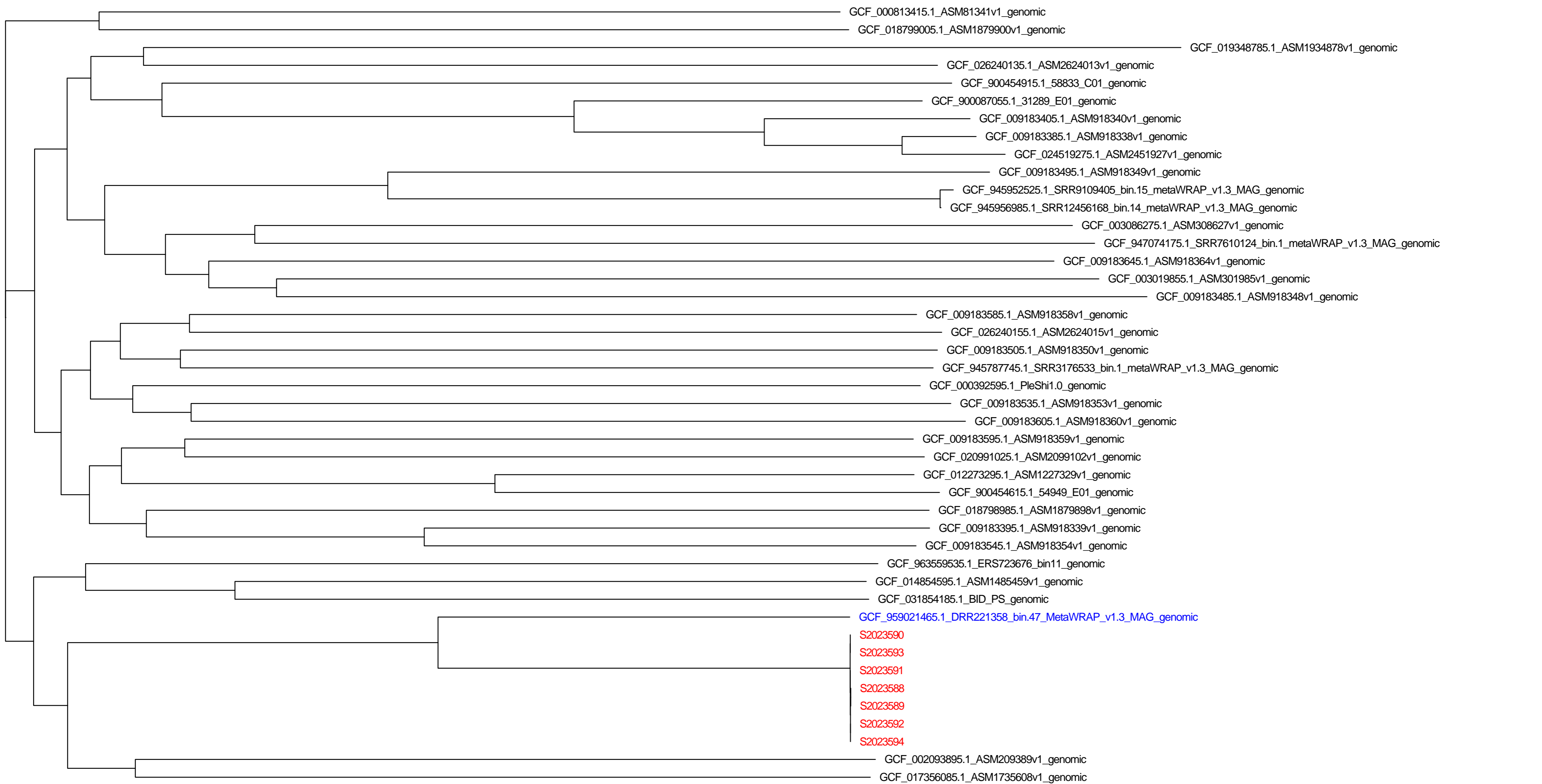

0.03

Supplement: S1 Raw images — (PDF) [file pone.0301623.s001.pdf]
