# Supplementary material for: Pathogenic characteristics of an aggregated diarrhea event caused by Plesiomonas shigelloides from stream
Source: PLoS One. 2024 Apr 4;19(4):e0301623. doi: 10.1371/journal.pone.0301623 (PMC10994385; doi:10.1371/journal.pone.0301623)
Supplement: Attachment — Submitted filename: PONE-D-24-02504.docm [file pone.0301623.s003.docm]

**PONE-D-24-02504**

1. Please verify, it is ‘Pseudomonas shigelliides’ or ‘Pseudomonas shigelloides’ or ‘Plesiomonas shigelloides’. In all the case it seems not ‘Pseudomonas’ See this article : Janda JM, Abbott SL, McIver CJ. Plesiomonas shigelloides Revisited. Clin Microbiol Rev. 2016;29(2):349-74. REALLY IT IS NOT ALLOWED TO MAKE THIS MISTAKES OF CONFUSION.
2. Please correct ‘Gram-negative’ not ‘gram-negative'. Verify this in all your article (see section discussion also)
3. In ‘diarrheal Escherichia coli’, the term ‘diarrheal’ should not be in italic.
4. In section ‘Sample collection and detection’, is this correct ‘pathogens, including Pseudomonas shigella’? please verify all your manuscript according to my first comment.
5. The term ‘MAC’ means ‘MacKonkey’ ? please write the full term without abbreviation, at least for the first time that you mention it.
6. Change the term’ Drug sensitivity test’ by ‘antimicrobial susceptibility test’
7. In all the manuscript, please verify punctuation and there are a lot of word not separated (especially in the section ‘antimicrobial susceptibility test’. Please never write the name of antibiotics with capital letters, write ‘ampicillin (AMP), ampicillin/sulbactam (AMS), cefotaxime (CTX), ceftazidime (CAZ), cefoxitin (CFX), Cefazolin (CFZ), imipenem (IMP), cefotaxime/clavulanic acid (CTX/C), ceftazidime/clavulanic acid (CAZ/C), amoxicilin/clavulanic acid (AMC), cefuroxime (CXM), cefepime (CPM), ceftazidime/avibactam (CZA), meropenem (MEM), ertapenem(ETP), ceftiofur (CEF), ciprofloxacin (CIP), nalidixic acid (NAL), gentamicin (Gen), amikacin(AMK), streptomycin (STR), tetracycline (TET), tigecycline (TIG), florfenicol (FFC), colistin (CT), Polymixin (PB), azithromycin (AZM), trimethoprim-sulfamethoxazole (SXT), and chloramphenicol (CHL). *E. coli* ATCC29522 was used as quality control strain’. THE ‘E. coli’ must be in italic.
8. The name of the enzyme used in PFGE must be in italic ‘*Spe*’ and ‘I’ not in italic, this is also for ‘Xba’
9. In ‘Salmonellaenterica serovar Braenderup’ make space between ‘Salmonella’ and enterica’ and write them in italic.
10. Please in section results, mention that *kpnH*, *rsmA*, *rsmA, adeF* and *CRP* are ‘antibiotic efflux pumps’ or ‘efflux resistance genes’. This could explain the multidrug resistance phenotype caused by each pump.
11. Please correct as follows:’.. Greenlees *et al.* [18]conducted a study that revealed a higher incidence of reported cases during the warmer months when water temperatures increase, facilitating the proliferation of plesiomonads through sewage contamination.’
12. Please in discussion, add 3 to 4 sentences describing the antibiotic efflux pumps (kpnH, rsmA, rsmA, adeF, CRP).
13. You said ‘which was submitted in June 2023,’ Please say from which country this strain was collected, and its source (food, water, patient???).
14. In conclusion, add ‘s” (several genes), so write ‘All isolates carry the same drug resistance genes and virulence genes except cgsE , and the homology between isolates is high.’
