# Supplementary material for: Pathogenic characteristics of an aggregated diarrhea event caused by Plesiomonas shigelloides from stream
Source: PLoS One. 2024 Apr 4;19(4):e0301623. doi: 10.1371/journal.pone.0301623 (PMC10994385; doi:10.1371/journal.pone.0301623)
Supplement: Attachment — Submitted filename: Response to Reviewers.docx [file pone.0301623.s004.docx]

**Dear Reviewer and Editor:**

We are very grateful to Reviewer for reviewing the paper so carefully, and hope that the correction will meet with approval. We have tried our best to improve and made some changes in the manuscript. Thank you very much for your comments and suggestions.

**Reviewer #1:**

**1.** Abstract, the content description is inaccurate. The author should state the time of event clearly, because there may be not only one aggregation diarrhea event in Huzhou,2023. It is suggested to describe it as “trace the origin of *P. shigelliides*” rather than “trace the origin of the pathogenic bacteria”. It is suggested to delete the "of the cases" in line 12.

**Response:** Thanks for your advice. The statements have been corrected. We will be happy to edit the text further based on comments from reviewers.

**2.**The English expression in the paper needs to be consistent for clarity. After the first appearance of "*Pseudomonas shigella*", "*P. shigelliides*" can be used as a substitute in the following. The same to "WGS" replace " whole genome sequencing".

**Response:** Thanks for your advice. These statements have been corrected in all the manuscript.

**3.** Sample collection and detection, it should be noted that which 22 pathogens can be detected by Filmarray GI test strip.

**Response:** Thanks for your advice. This Filmarray GI test strip include *Campylobacter* (*C. jejuni/C. coli/ C. upsaliensis)*, *Clostridium difficile* (toxin A/B), ***Plesiomonas shigelloides***, *Salmonella*, *Vibrio (V. parahaemolyticus/V. vulnificus/ V. cholerae)*, *Yersinia enterocolitica*, EAEC, EPEC, ETEC, STEC, *E. coli* O157, EIEC, *Cryptosporidium*, *Cyclospora cayetanensis, Entamoeba histolytica, Giardia lamblia* (also known as *G. intestinalis* and *G. duodenalis*), Adenovirus F 40/41, Astrovirus, Norovirus GI/GII, Rotavirus A, Sapovirus (Genogroups I, II, IV, and V).


**4.** Sample collection and detection, which paper or methods were referenced for the isolation and culture. *P. shigelliides* does not have standards for isolation now.

**Response:** Thanks for your advice. Samples used to test for *Plesiomonas shigelloides* should be collected and handled the same as those for any routine enteric pathogens. According to reference 3 to isolation and culture.

**5.** Sequence analysis, the genome sequences of the 7 *P. shigelloides* isolates should be submitted to GenBank/DDBJ/ENA and acquired the accession numbers.

**Response:** Thanks for your advice. The genome sequences of the 7 *P. shigelloides* isolates had been submitted to GenBank. The accession numbers are [JAZHQW000000000](https://www.ncbi.nlm.nih.gov/nuccore/JAZHQW000000000), [JAZHQX000000000](https://www.ncbi.nlm.nih.gov/nuccore/JAZHQX000000000), [JAZHQY000000000](https://www.ncbi.nlm.nih.gov/nuccore/JAZHQY000000000), [JAZHQZ000000000](https://www.ncbi.nlm.nih.gov/nuccore/JAZHQZ000000000), [JAZHRA000000000](https://www.ncbi.nlm.nih.gov/nuccore/JAZHRA000000000), [JAZHRB000000000](https://www.ncbi.nlm.nih.gov/nuccore/JAZHRB000000000), [JAZHRC000000000](https://www.ncbi.nlm.nih.gov/nuccore/JAZHRC000000000).

**6.** Discussion, in paragraph 1, it is recommended to add a description of local distribution of *P. shigelliides* formerly and the measures taken by the CDC after this diarrhea aggregation event.

**Response:** Thanks for your advice. These statements have been added in discussion.

**7.** Discussion, antibiotic resistance, especially multidrug resistance(MDR), is becoming increasingly severe. It is suggested to add discussion of MDR in paragraph 3.

**Response:** Thanks for your advice. We agree with you and added some sentences about MDR in discussion paragraph 3.

**8.** Discussion, in paragraph 6,this study found the most closely related strain was GCF_959021465.1_DRR221358_bin.47_MetaWRAP_v1.3_MAG_genomic. But there is no description of the differences in the number of SNP variation. It is suggested to supplement the explanation in the paper.

**Response:** Thanks for your advice. Although this reference sequence is the most closely related with our study, there are still 18225 differences in SNP sites. We cannot assume that they are homologous. These statements have been added in discussion.

**Reviewer #2:**

1. Please verify, it is ‘Pseudomonas shigelliides’ or ‘Pseudomonas shigelloides’ or ‘Plesiomonas shigelloides’. In all the case it seems not ‘Pseudomonas’ See this article : Janda JM, Abbott SL, McIver CJ. Plesiomonas shigelloides Revisited. Clin Microbiol Rev. 2016;29(2):349-74. REALLY IT IS NOT ALLOWED TO MAKE THIS MISTAKES OF CONFUSION.

**Response:** We thank the reviewer of pointing out this issue. We apologize for the confusion generated by the manuscript. It is *Plesiomonas shigelloides* in this article. We had replaced all ‘*Pseudomonas shigelliides*’ with ‘*Plesiomonas shigelloides*’.

2. Please correct ‘Gram-negative’ not ‘gram-negative'. Verify this in all your article (see section discussion also)

**Response:** We are very sorry for our incorrect writing and it is rectified.

3. In ‘diarrheal Escherichia coli’, the term ‘diarrheal’ should not be in italic.

**Response:** Thanks for your advice. We are very sorry for our incorrect writing and it is rectified.

4. In section ‘Sample collection and detection’, is this correct ‘pathogens, including Pseudomonas shigella’? please verify all your manuscript according to my first comment.

**Response:** Sorry, we had replaced ‘*Pseudomonas shigelliides*’ with ‘*Plesiomonas shigelloides*’ in my manuscript.

5. The term ‘MAC’ means ‘MacKonkey’ ? please write the full term without abbreviation, at least for the first time that you mention it.

**Response:** Thanks for your advice. The term ‘MAC’ means ‘MacKonkey’. We had changed to the full term.

6. Change the term’ Drug sensitivity test’ by ‘antimicrobial susceptibility test’.

**Response:** Thanks for your advice. We had replaced ‘Drug sensitivity test’ with ‘antimicrobial susceptibility test’ .

7. In all the manuscript, please verify punctuation and there are a lot of word not separated (especially in the section ‘antimicrobial susceptibility test’. Please never write the name of antibiotics with capital letters, write ‘ampicillin (AMP), ampicillin/sulbactam (AMS), cefotaxime (CTX), ceftazidime (CAZ), cefoxitin (CFX), Cefazolin (CFZ), imipenem (IMP), cefotaxime/clavulanic acid (CTX/C), ceftazidime/clavulanic acid(CAZ/C), amoxicilin/clavulanic acid (AMC), cefuroxime (CXM), cefepime (CPM), ceftazidime/avibactam (CZA), meropenem (MEM), ertapenem(ETP), ceftiofur (CEF), ciprofloxacin (CIP), nalidixic acid (NAL), gentamicin (Gen), amikacin(AMK), streptomycin (STR), tetracycline (TET), tigecycline (TIG), florfenicol (FFC), colistin (CT), polymixin (PB), azithromycin (AZM), trimethoprim-sulfamethoxazole (SXT), and chloramphenicol (CHL).*E. coli* ATCC29522 was used as quality control strain’. THE ‘E. coli’ must be in italic.

**Response:** Thanks for your advice. We had verified punctuation in all the manuscript and edited the capital letters of the antibiotics. The ‘*E. coli*’ was used in italic.

8. The name of the enzyme used in PFGE must be in italic ‘*Spe*’ and ‘I’ not in italic, this is also for ‘Xba’

**Response:** We are very sorry for our incorrect writing and it is rectified.

9. In ‘Salmonellaenterica serovar Braenderup’ make space between ‘Salmonella’ and enterica’ and write them in italic.

**Response:** Thanks for your advice and the statement have been corrected.

10. Please in section results, mention that *kpnH*, *rsmA*, *rsmA, adeF* and *CRP* are ‘antibiotic efflux pumps’ or ‘efflux resistance genes’. This could explain the multidrug resistance phenotype caused by each pump.

**Response:** Thanks for your advice. *kpnH*, *rsmA*, *rsmA, adeF* and *CRP* are ‘antibiotic efflux pumps’. These were added in section results.

11. Please correct as follows:’.. Greenlees *et al.* [18]conducted a study that revealed a higher incidence of reported cases during the warmer months when water temperatures increase, facilitating the proliferation of plesiomonads through sewage contamination.’

**Response:** Thanks for your advice. We had corrected as follows: ‘.. Greenlees *et al.* [18]conducted a study that revealed a higher incidence of reported cases during the warmer months when water temperatures increase, facilitating the proliferation of plesiomonads through sewage contamination.’

12. Please in discussion, add 3 to 4 sentences describing the antibiotic efflux pumps (kpnH, rsmA, rsmA, adeF, CRP).

**Response:** Thanks for your advice. We had added some sentences describing the antibiotic efflux pumps in discussion.

13. You said ‘which was submitted in June 2023,’ Please say from which country this strain was collected, and its source (food, water, patient???).

**Response:** The RefSeq of this strain was collected from United Kingdom and its source was human feces. These were added to the manuscript. We can found information from ‘https://www.ncbi.nlm.nih.gov/nuccore/CAUEVM000000000.1’

14. In conclusion, add ‘s” (several genes), so write ‘All isolates carry the same drug resistance genes and virulence genes except cgsE , and the homology between isolates is high.’

**Response:** Thanks for your advice. We are very sorry for our incorrect writing and it is rectified.
